# Cytotoxicity and Oxidative Stress Induced by Technology-Critical Elements *versus* Traditional Metal Contaminants: An *In Vitro* Bioassay Study

**DOI:** 10.1021/acs.est.4c09710

**Published:** 2025-01-06

**Authors:** Anna Qvarforth, Anna Augustsson, Michelle von Ehr, Geeta Mandava, Ilia Rodushkin, Emma Engström, Steffen Eisele, Johan Lundqvist

**Affiliations:** † Department of Animal Biosciences, Swedish University of Agricultural Sciences, Box 7028, SE-750 07 Uppsala, Sweden; ‡ Department of Biology and Environmental Science, Linnaeus University, Stuvaregatan 4, 392 31 Kalmar, Sweden; § ALS Laboratory Group, ALS Scandinavia AB, Aurorum 10, 977 75 Luleå, Sweden; ∥ Division of Geosciences and Environmental Engineering, Luleå University of Technology, Laboratorievägen 14, 971 87 Luleå, Sweden

**Keywords:** emerging contaminants, metals, health risks, toxicity, reporter genes

## Abstract

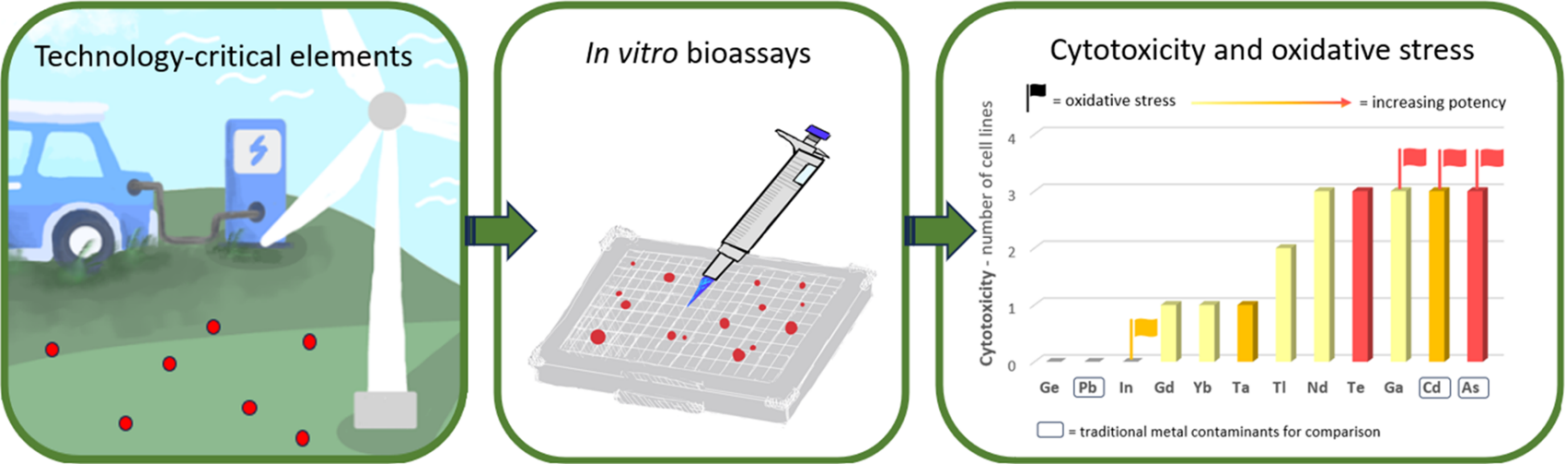

Technology-critical elements (TCEs), essential in emerging technologies, are increasingly finding their way into our environment, raising concerns about their sparsely studied behavior and toxicity. To contribute insights into the toxicological aspects, we employed *in vitro* bioassays to investigate the possible cytotoxic effects in four representative cell lines (AR-EcoScreen GR-KO-M1, DR-EcoScreen, MCF7AREc32, VM7Luc4E2) and the potential to induce oxidative stress via the nuclear factor erythroid 2-related factor 2 (Nrf2) pathway for a number of these elements. Nine TCEs, three rare-earth elements (REEs: Gd, Nd, Yb) and six less-studied TCEs (LSTCEs: Ga, Ge, In, Ta, Te, Tl), were selected for this study, along with three well-studied traditional metal contaminants (TMCs: As, Cd, Pb) for comparison. Among the 12 studied elements, nine showed signs of inducing cytotoxicity: As, Cd, Ga, Nd, and Te in three out of the four studied cell lines and Gd, Ta, Tl, and Yb in one to two cell lines. Tellurium repeatedly exhibited the highest potency. The TCEs Ga and In, similar to As and Cd, also demonstrated the potential to induce oxidative stress. The results of this study suggest that some TCEs may potentially cause adverse health effects similar to As and Cd, thus prompting further investigations.

## Abstract 2

This study contributes valuable new knowledge to the field of environmental toxicology, focusing on the propensity of technology-critical elements to induce cytotoxicity and oxidative stress.

## 1 Introduction

Technology-critical elements (TCEs) are metal(loid)s, whose use and extraction have skyrocketed over the past decades due to their central role for our green transition and for ensuring the EU’s green and digital future. According to the European COST Action TD1407: Network on Technology-critical elements (NOTICE), the TCE group comprises (1) most rare-earth elements (REEs); (2) the platinum group elements (PGEs); and (3) another seven elements; gallium (Ga), germanium (Ge), indium (In), niobium (Nb), tantalum (Ta), tellurium (Te), and thallium (Tl). The latter subgroup is sometimes referred to as the less studied TCEs (LSTCEs).^1−3^

These elements are currently used in, e.g., renewable energy systems, electric- and hybrid vehicles, electronics, energy-efficient lightening, metallurgy, defense systems, equipment used in communication, and medicine.^4−10^ While the green transition and TCE-dependent emerging technologies bring significant benefits, they also carry potential risks. The TCEs naturally, and still mostly, occur in ultra trace concentrations in our environment. Recently, however, increasing concentrations have been observed, particularly near industries, but also in more rural environments; in soil, ground- and surface water, sediments, glaciers, and biota.^4,9,11−13^ With the steep rise in demand,^14−16^ further increases in environmental concentrations are expected. However, the risks we face in such scenarios remain largely unknown, as there are significant gaps in our understanding of the TCEs’ environmental behavior, routes of human exposure, and perhaps most crucially: their toxicity.^17^

The negative effects of many other metal(loid)s, like arsenic (As), cadmium (Cd), chromium (Cr), lead (Pb), and mercury (Hg), are well-known and include both acute and chronic effects, like neurogenerative disorders, kidney failure, cardiovascular diseases, osteoporosis, lung diseases, and cancer.^18^ In that sense, metals make a significant contribution to the global burden of disease, and it would be surprising if there were not members of the TCE group that shared at least some of these other metals’ potential to induce negative health effects. While the toxic properties and pathways of the TCEs are poorly studied, there are still some previous research which has indicated or confirmed certain toxic effects, primary following animal experiments,^19−24^ but also in humans in occupational settings.^23,25−27^ Examples of observed effects include liver, respiratory, and kidney damages, neurological impairments, gestational diabetes mellitus, genotoxicity, bone alterations, fibrotic tissue injury, male sterility, and skin and eye irritations for the REEs;^20,28−33^ gastrointestinal disorders, neurological damages, hair loss, heart failure, internal bleeding, paralysis, collapse, and death for Tl;^34−36^ and effects on kidneys and the respiratory system for Ga and Ge.^22,37^

For effects like those listed above, it is essential to understand the underlying cellular mechanisms to fully grasp the toxicity associated with a specific element.^38^ For traditional metal contaminants, both oxidative stress and cytotoxicity are key factors in disease development. This has been observed, e.g., for As,^39−42^ Cd,^42−44^ and Pb.^45−47^ Assessing the induction of oxidative stress and cytotoxicity is thus crucial for evaluating an element’s potential contribution to disease.

In this context, the utilization of *in vitro* reporter gene bioassays offers valuable opportunities to elucidate, e.g., specific cellular oxidative stress mechanisms, where reporter gene assays incorporate a gene encoding a readily detectable protein downstream the oxidative stress response.^38^ To the best of our knowledge, no previous study has encompassed this type of analysis for the increasingly used TCEs. Therefore, the aim of this study was to employ *in vitro* reporter gene bioassays to assess the potential of selected TCEs to induce oxidative stress through interaction with the Nuclear factor erythroid 2-related factor 2 (Nrf2) pathway. Additionally, considering also the significance of cytotoxicity, this aspect was investigated in four different cell lines.

## 2 Materials and Methods

### 2.1 Studied Metals and Method Overview

From the group of rare-earth elements (REEs), one representative with low molecular weight (Nd) was selected, one with medium (Gd), and one with high molecular weight (Yb). The inclusion of these three REEs was also motivated by their widespread use and indications of toxicity from previous studies.^20,29,31^ From the heterogeneous group referred to as LSTCEs, all elements except Nb were chosen for investigation. This element was excluded since it has consistently demonstrated low toxicity with high LD_50_ values in previous studies.^48,49^ Finally, with the aim of contextualizing the results in comparison to more well-known elements, three traditional metal contaminants (TMCs), namely As, Cd, and Pb, were also included.

To test the cytotoxicity and oxidative stress induction of these elements, *in vitro* bioassays were conducted at the Swedish University of Agricultural Sciences (SLU) during the spring of 2023. The assays are summarized in Table 1. Cytotoxicity was assessed by measuring decreased cell viability across four different cell lines: AR-EcoScreen GR-KO-M1, DR-EcoScreen, MCF7AREc32, and VM7Luc4E2. These distinct and commonly used^38^ cell lines were selected to provide a comprehensive evaluation of the research question. The MCF7AREc32 and VM7Luc4E2 lines, derived from human sources, offer insights into human cellular processes, while the AR-EcoScreen GR-KO-M1 and DR-EcoScreen lines, sourced from Chinese hamster ovary and mouse hepatoma cells respectively, represent different organ systems in animal models. This diverse selection of cell types captures variations in sensitivity and biological response, thereby enhancing the relevance of the findings. Oxidative stress was evaluated through Nrf2 induction in the MCF7AREc32 cell line. In this test, *tert*-butylhydroquinone (tBHQ) was used as a reference compound, as is common practice in oxidative stress assays due to its role in activating the Nrf2 pathway.^38^ Absorbance measurements, detailed in the Supporting Information, were used to reflect the activities for the studied end points. A substance was considered cytotoxic when the cell viability was <80% relative to the vehicle. Further, the potency to induce cytotoxicity was assessed from interpolated values of 70% inhibitory concentrations, IC_70_. Inhibitory concentrations of 70%, commonly used in other studies as well, were chosen to ensure a value clearly below 100% – avoiding the classification of normal variation as cytotoxic–while still being high enough to maintain method sensitivity. For oxidative stress, an effective concentration at an induction ratio of 1.5 (EC_IR1.5_) was used to differentiate Nrf2 induction activity. Lower values of these two metrics, i.e., the IC or the EC_IR1.5_, imply activity already at lower concentrations and thus, higher potency.

**Table 1:** Method Summary^a^

main target effect	measured response	cell lines	reference compound	cutoff value for cytotoxicity/bioactivity
cytotoxicity	reduction in cell viability	MCF7AREc32	-	<80% cell viability vs vehicle control, i.e., 20% reduction
		AR-EcoScreen GR-KO-M1		
		VM7Luc4E2		
		DR-EcoScreen		
oxidative stress	Nrf2 activity	MCF7AREc32	*tert*-butylhydroquinone (*t*BHQ)	1.5-fold increase in activity vs vehicle control

a The response in the MCF7AREc32, AR-EcoScreen GR-KO-M1, and DR-EcoScreen cell lines was analyzed with the MTS assay, and the response in the VM7Luc4E2 cell line was analyzed with the ATPase assay.

### 2.2 Bioassays

The assays utilized to evaluate cytotoxicity were the MTS and ATPase assays.^50,51^ To assess the elements’ potential for inducing oxidative stress, the evaluation of Nrf2 induction was specifically conducted in the MCF7AREc32 cell line, a commonly used cell line for *in vitro* studies of oxidative stress.^38^ The concomitant screening for cytotoxicity in this cell line served the purpose of ensuring that the investigated Nrf2 interaction was studied at noncytotoxic concentrations. If not secured, cytotoxicity can mask the actual results in the activity assay.^38^ A detailed description of how each individual assay was conducted can be found in the Supporting Information.

The elements were introduced to the cell cultures via commercially available stock solutions, typically diluted in HNO_3_ (the vehicles of all elements are provided in Table 2). To enable an initial hazard identification, which this study can be seen as, it is necessary to be within the concentration range where effects clearly begin to be observable, even if these concentrations are higher than those we are currently exposed to in a present-day scenario. The goal was therefore to start from a concentration as high as 10,000 mg/L for each solution, which would result in a maximum cell exposure of 100 mg/L in the bioassay experiments after dilution. This maximum exposure concentration is limited by the requirement that the growth medium cannot be diluted beyond 1% without affecting its functional nutrient composition. However, for some of the elements only 1000 mg/L stock solutions were available, leading to a maximum exposure of 10 mg/L. Further concentrations were established using 5-fold dilutions of the maximum levels. In the end, the evaluated concentrations for Ga, Nd, Yb, and Pb were: 100, 20, 4.0, 0.80, 0.16, 0.032, 0.0064, and 0.00128 mg/L. For As, Cd, Gd, Ge, In, Ta, Te, and Tl, they were: 10, 2.0, 0.40, 0.080, 0.016, 0.0032, 0.00064, and 0.000128 mg/L. All measurements were made on quadruplicates of samples. The cell exposure time was 24 h. As negative controls the vehicles in which the elements were dissolved were used, i.e., HNO_3_ at different concentrations for all elements except Ge and Ta, for which deionized water was used.

**Table 2:** Technology-Critical Elements (TCEs) and Traditional Metal Contaminants Included in the Study

element	Gd^a^	Nd^a^	Yb^a^	Ga^b^	Ge^b^	In^b^	Ta^b^	Te^b^	Tl^b^	As^c^	Cd^c^	Pb^c^
vehicle	2% HNO_3_	5% HNO_3_	2–5% HNO_3_	5% HNO_3_	H_2_O	2–5% HNO_3_	H_2_O	5% HNO_3_	2–3% HNO_3_	2% HNO_3_	2% HNO_3_	2% HNO_3_
CAS-nr	7440–54–2	7440–00–8	7440–64–4	7440–55–3	7440–56–4	7440–74–6	7440–25–7	13494–80–9	7440–28–0	7440–38–2	7440–43–9	7439–92–1
manu-facturer	Avantor	CPAchem	ARISTAR, Avantor	Thermo Fisher Scientific	SPEX CertiPrep LLC	ARISTAR, Avantor	Supelco	Thermo Fisher Scientific	Supelco, Merck	Agilent	Agilent	Agilent

a Rare-earth elements.

b Less studied TCEs.

c Traditional metal contaminants.

### 2.3 Data Evaluation

Results from the cytotoxicity (= cell viability) tests were all normalized against the responses of the vehicles/negative controls, which were set to 100%, and samples giving >20% reduction in cell viability were considered cytotoxic. Standard curves were generated through a nonlinear regression sigmoidal curve fit employing the GraphPad Prism 10.1.0 Software. The inhibitory concentration resulting in 70% response (IC_70_) relative to the control, were subsequently interpolated from the regression curve, following the methodology outlined by Escher et al.^52^ In some cases, where the obtained data did not allow for the interpolation of IC_70_, a value of IC_80_ value was interpolated instead. In other cases, and to facilitate comparison with previous studies discussing potency in terms of IC_50_ values, these values were interpolated when the data set permitted. When evaluating the oxidative stress response, the activities were again normalized against the vehicle controls. The standard curves, generated in GraphPad Prism, underwent linear regression, and the effective concentration for the induction ratios (EC_IR_) of 1.5 (EC_IR1.5_) was extrapolated from Nrf2 activity, considering the absence of maximum responses, such as in cases with receptor saturation.^52^ An EC_IR_ of 1.5 is commonly considered a suitable benchmark for a significant effect, well above the limit of detection.^52^

## 3 Results and Discussion

### 3.1 Overview of Results and Human Relevance

An overview of the results is presented in Table 3, where green indicates no activity and red denotes the highest potency, corresponding to the lowest IC_70_ or EC_IR1.5._ values. Cytotoxicity was observed in all cell lines and for 9 of the investigated elements (As, Cd, Ga, Gd, Nd, Ta, Te, Tl, Yb). Kamiloglu et al.^53^ emphasize the importance of conducting multiple assays, as there is stronger evidence of general cytotoxicity when consistent results are observed across various assays and cell lines, as observed for As, Cd, Ga, Nd, and Te. Oxidative stress, inferred from increased Nrf2 activity, was observed for two TCEs (Ga, In), with particularly pronounced effects for Ga, as well as for As and Cd.

**Table 3:**
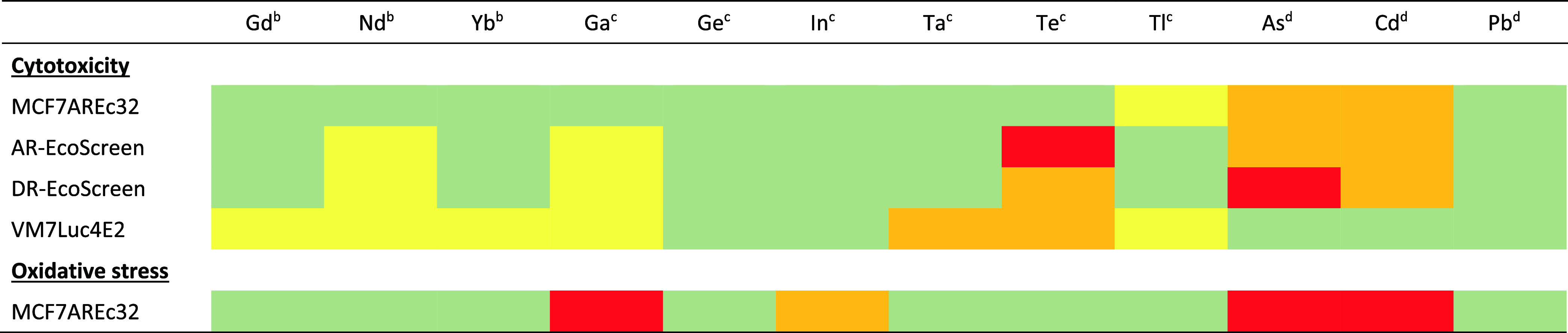
Overview of the Results Obtained from the Four Cytotoxicity Assays and the Oxidative Stress Assay^a^

a Green coloring indicates the absence of cytotoxicity/triggering of oxidative stress. Responses giving values of IC_70_ or EC_IR1.5_ in the range of 11–100 mg/L, or those with an unclear concentration–response relationship, are marked in yellow; those between 1 and 10 mg/L in orange; and those below 1 mg/L (highest potency) in red.

b Rare-earth elements.

c Less studied TCEs.

d Traditional metal contaminants.

Results for the positive controls, demonstrating the functionality of the assays, can be found in the Supporting Information, Figure S1A–B.

*In vitro* bioassays are particularly effective in identifying the potential of new substances to induce specific responses, serving as a useful screening tool in the early stages of the hazard identification. However, the concentrations required to elicit statistically significant responses in these tests provide little insight into the levels (e.g., in blood) that are associated with a specific probability of disease in a human population. Consequently, it is difficult to ascertain whether a detected response for a novel compound in an *in vitro* bioassay is relevant at the physiological concentrations encountered *in vivo*. To facilitate a preliminary assessment of the real-life relevance of a detected response, reference elements with well-documented effects in human populations and established dose–response relationships can be included in the experiment. Therefore, the results and discussion in this TCE-focused paper will be grounded in the findings for As, Cd, and Pb.

### 3.2 Cytotoxicity

**Figure 1:**
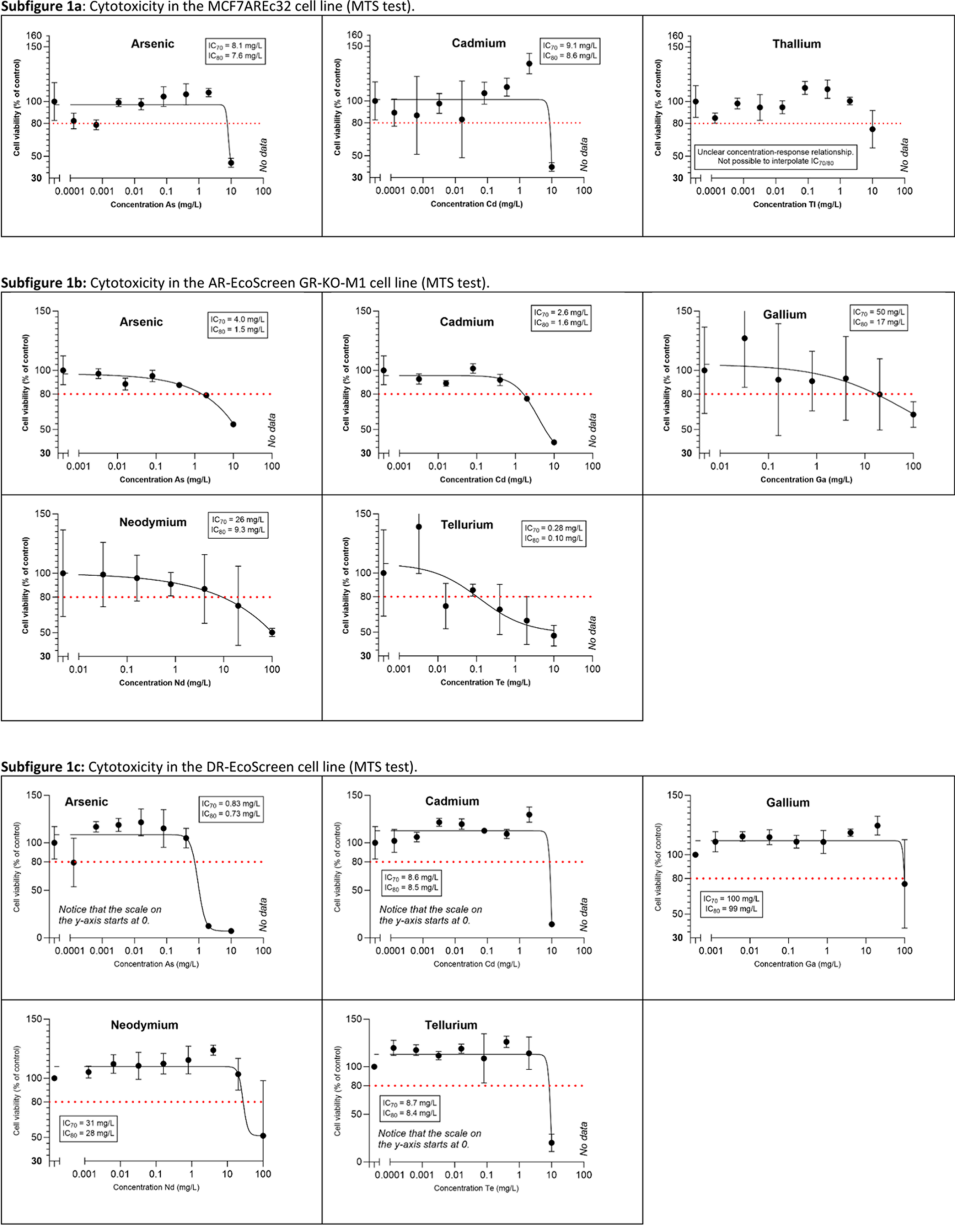

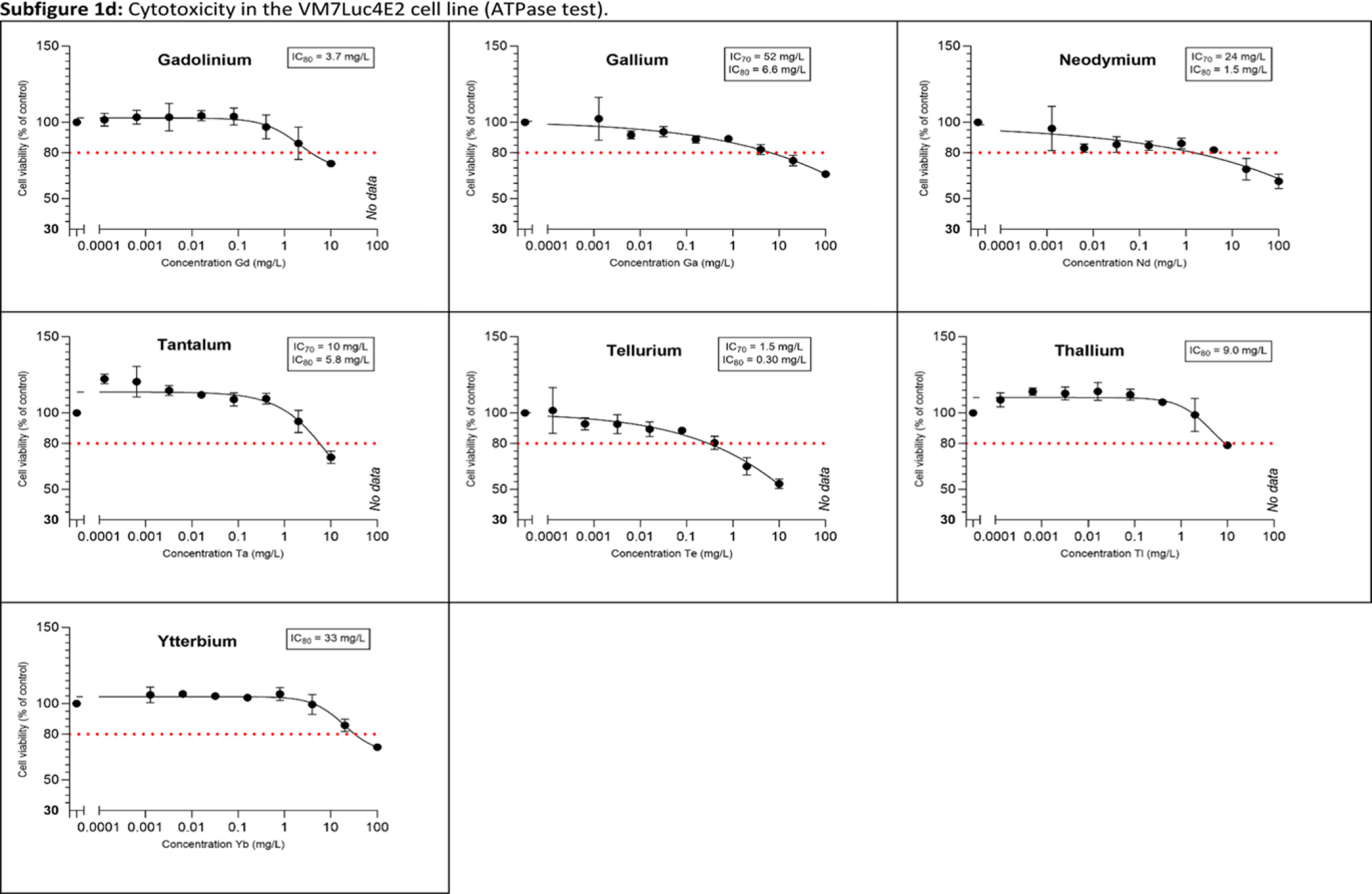
Concentration–response curves for the elements showing cytotoxicity in the four different cell lines. The red dotted line indicates the cutoff value for cytotoxicity, defined as cell viability <80% compared to the vehicle control. For each assessed concentration, data are presented as the mean value ± standard deviation (*n* = 4). Note that the scales on the *y*-axes differ. The corresponding curves for all elements, including those that did not show signs of cytotoxicity, are presented in the Supporting Information, Figures S2–S5.

Figure 1 shows the concentration–response curves for the elements with observed cytotoxicity, defined as cell viabilities below 80%. Complete data for all elements and cell lines can be found in the Supporting Information, Figures S2–S5.

#### 3.2.1 Traditional Metal Contaminants

Of the tested TMCs, cytotoxicity was identified in the MCF7AREc32, AR-EcoScreen, and DR-EcoScreen cell lines for both As and Cd (Figure 1). For As, the interpolated IC_70_ values (mg/L) ranged from 0.83 (DR-EcoScreen) to 8.1 (MCF7AREc32), and for Cd from 2.6 (AR-EcoScreen) to 9.1 (MCF7AREc32).

For As, interpolated IC_50_ values, converted to mmol/L to facilitate comparison with existing literature, ranged from 0.014 to 0.17 mmol/L in the DR-EcoScreen and AR-EcoScreen cell lines, respectively. This range aligns with the IC_50_ value of 6.7 mg/L (0.090 mmol/L) reported in HepG2 hepatocarcinoma cell lines by Cordier et al.^54^ Our results for Cd also show reasonable concordance with previous studies using cell-based assays to assess cytotoxicity in short-term or acute settings, although our findings fall on the higher end of the data range reported in the scientific literature. The Cd IC_50_ values from our study ranged between 0.052 mmol/L (AR-EcoScreen) and 0.087 mmol/L (MCF7AREc32), while literature values span from 0.001 to 0.080 mmol/L.^54−56^ Sauvant et al.^56^ reported Cd IC_50_ values between 0.009 and 0.04 mmol/L across six different assays on the L-929 murine fibroblast cell line, with a broader literature compilation in the same article showing values from 0.0010 to 0.080 mmol/L. A study by Al-Ghafari et al.^55^ found IC_50_ values of 0.032 mmol/L (MTT assay) and 0.063 mmol/L (LDH assay) for Cd in human bone osteoblasts, while Cordier et al.^54^ reported an IC_50_ of 0.43 mg/L (0.0038 mmol/L) for Cd. Thus, our findings for As and Cd are overall consistent with those of earlier studies, despite differences in the cell lines used.

For Pb, no cytotoxicity was observed in any of the cell lines used in our study (Figures S2–S5), despite substantial evidence from previous research demonstrating the element’s cytotoxic properties.^45−47^ The most plausible explanation is that the maximum concentration tested in our study, 100 mg/L or 0.48 mmol/L, was too low, as there are similar studies conducted in the past which have observed significant effects only at relatively high concentrations. In the Sauvant et al.^56^ study, for example, IC_50_ values for Pb varied between 98 mg/L (0.47 mmol/L) and 580 mg/L (2.8 mmol/L). There are, however, also some examples of studies which have reached lower IC_50_ values, like that of Al-Ghafari et al.^55^ who reported IC_50_ values of 0.055 mmol/L (MTT) and 0.079 mmol/L (LDH) in human bone osteoblasts for Pb. Additionally, the literature compilation in the Sauvant et al.^56^ article reports Pb IC_50_ values of 0.10 to 2.7 mmol/L in a variety of cell types.

Building on the extensive toxicity data for Pb, with Pb-related diseases having a significant impact on the general human population worldwide, it is clear that the concentration ranges associated with cytotoxicity in 24 h *in vitro* bioassays far exceed those typically relevant in biological samples. The World Health Organization assesses that Pb exposure globally accounts for 21.7 million disability-adjusted life years (DALYs) lost, 30% of the burden of idiopathic intellectual disability, 4.6% of cardiovascular disease, and 3% of chronic kidney disease.^57^ Yet, human blood Pb (B–Pb) concentrations are usually much lower than those required for a distinct response in *in vitro* bioassays, even in highly exposed individuals.^45,46^ The U.S. Centers for Disease Control and Prevention (CDC), for instance, uses a B–Pb value of 3.5 μg/dL (∼0.00017 mmol/L) to identify children at the 97.5th percentile.^58^ In adults, chronic kidney disease is the effect observed at the lowest B–Pb levels, with EFSA^46^ estimating a 1% increased risk at approximately 15 μg/L (0.000072 mmol/L). For children, neurotoxicity is the most critical concern, with a 1% increased risk of intellectual impairment occurring at around 12 μg/L in B–Pb (0.000058 mmol/L) according to the same source. Although mechanisms other than cytotoxicity may primarily drive these conditions, this example with Pb highlights how concentrations from *in vitro* bioassays poorly match the internal doses linked to manifested diseases.

#### 3.2.2 Gallium, Neodymium, and Tellurium

Cytotoxic effects were most clearly observed for the TCEs Ga, Nd, and Te (of which Nd is a REE), found in 3 out of the 4 tested cell lines; both the AR- and DR-EcoScreen cell lines and the VM7Luc4E2 cell line (Figure 1b–d). Out of these, Te consistently showed the lowest IC_70_ values (0.28, 8.7, and 1.5 mg/L, respectively, equaling 2.2 × 10^–3^, 0.068 and 0.012 mmol/L), in many cases even lower than the corresponding values for As and Cd. Following animal studies revealing neurotoxic effects,^59−61^ Roy and Hardej^62^ investigated and found that both an organic form of Te (diphenyl ditelluride, DPDT) and an inorganic form (tellurium tetrachloride, TeCl_4_) could induce cytotoxicity in rat hippocampal astrocytes. In experiments with human promyelocytic cells (line HL-60), Sailer^63^ also discovered that organotellurium compounds could induce apoptosis in a time- and dose-dependent manner.

For Ga, the IC_70_ values in our study were interpolated to 50, 100, and 52 mg/L (or 0.72, 1.4, and 0.75 mmol/L), and for Nd to 26, 31, and 24 mg/L (0.18, 0.21, and 0.17 mmol/L). Compared to both the TMCs and Te, the literature data for Ga and Nd is more limited. The mechanisms of action proposed for Ga thus far involve competition with iron for transferrin binding, subsequently causing cell destruction,^64−66^ making our results considering cytotoxicity expected. The same applies to Nd, where Ahmad et al.^67^ have observed cell death following Nd (Nd_2_O_3_) exposure to liver (HepG-2) and lung (A-549) cancer cells. Chen et al.^68^ also discovered that exposure to Nd_2_O_3_ activated the apoptosis pathway in zebrafish embryos and caused toxicity and abnormal development of the cardiac and cerebrovascular systems. Further, Huang et al.^69^ observed cytotoxicity in rat NR8383 alveolar macrophages following exposure of the same compound. No noticeable cytotoxicity was observed in the Huang et al.^69^ study at concentrations up to 6.25 mg/L, but thereafter, it increased dose-dependently up to the highest tested concentration of 200 mg/L.

#### 3.2.3 Thallium

Cytotoxicity for Tl was observed in the MCF7AREc32 cell line (Figure 1a), albeit less marked than for As and Cd, and neither an IC_70_ nor IC_80_ value could be calculated because of the unclear concentration–response relationship. Thallium-induced cytotoxicity was more evident in the VM7Luc4E2 cell line (Figure 1d), which showed no cytotoxic response to any of the TMCs. The IC_80_ for Tl in this cell line was 9.0 mg/L (0.044 mmol/L). Several researchers emphasize that Tl’s toxicity is as severe as that of As, Cd, Hg, and Pb,^70−72^ but in general, other explaining mechanisms than cytotoxicity (and oxidative stress) have been proposed. In particular, it has been suggested that chemical similarities with the essential nutrient potassium (K) allow Tl to utilize and subsequently disturb metabolic processes involving K.^70,73^ The two elements’ similarity also suggests that they pass through cell membranes similarly, and Tl ions have also been suggested to disturb the function of K ions in cardiac contraction mechanisms.^34^ However, our data now adds to this previous understanding, indicating that cell death can potentially be an additionally important mechanism contributing to Tl′s toxicological profile.

#### 3.2.4 Gadolinium, Tantalum, and Ytterbium

The elements Gd, Ta, and Yb were found to induce cytotoxic effects only in the VM7Luc4E2 cell line. This cell line was the only one to exhibit cytotoxic responses to all TCEs that displayed cytotoxicity, while showing no cytotoxicity to any of the TMCs. The achieved data set did not allow for interpolation of IC_70_ values though (except for Ta), but interpolated IC_80_ values increased in the order Gd (3.7 mg/L or 0.024 mmol/L) < Ta (5.8 mg/L or 0.032 mmol/L) < Yb (33 mg/L or 0.19 mmol/L), according to Figure 1d.

Very little research has been done previously on the potential cytotoxicity and oxidative stress associated with Gd, Ta and Yb. There is, however, a study by Xia et al.^74^ where they exposed rat cortical neurons to GdCl_3_*in vitro*, and also observed cytotoxic responses. Wang et al.^75^ evaluated the effects of Ta nanoparticles on the mouse osteoblast cell line MC3T3-E1 and found that these cells could be damaged through cytotoxicity and oxidative stress. As for Yb, another *in vitro* study, conducted on bone marrow stromal cells, showed a cytotoxic effect after Yb^3+^ exposure, particularly at the highest concentration of 1 mmol/L (∼170 mg/L).^76^

#### 3.2.5 Germanium and Indium

Neither Ge nor In exhibited any cytotoxicity in any cell line. However, in general, cell viability decreased with increasing concentrations of these elements, and the cutoff value of <80% cell viability compared to the vehicle control would likely be crossed at higher concentrations (Figures S2–S5).

Although the results from our studied cell lines did not suggest cytotoxicity as an underlying mechanism of toxicity, there are a few other examples implying that it could still be a concern. In a study by Lin et al.,^77^ mitochondrial damage was proposed to precede neurological damage following *in vitro* experiments with GeO_2_ in a mouse neuroblastoma cell line, Neuro-2A. Similarly, when epithelial cells (16HBE) and macrophages (RAW264.7) were exposed to indium oxide nanoparticles *in vitro*, cytotoxic responses were detected.^78^ On the other hand, only a low level of cytotoxicity of GeCl_4_ in *in vitro* studies with immortalized human skin keratinocytes and mouse fibroblasts (HaCaT and Balb/c 3T3 cell lines) was reported in a study by Sabbioni et al.^79^ This suggests that while Ge may have some cytotoxic potential, it might not be particularly pronounced, and the same could be true for In.

### 3.3 Oxidative Stress

**Figure 2:**
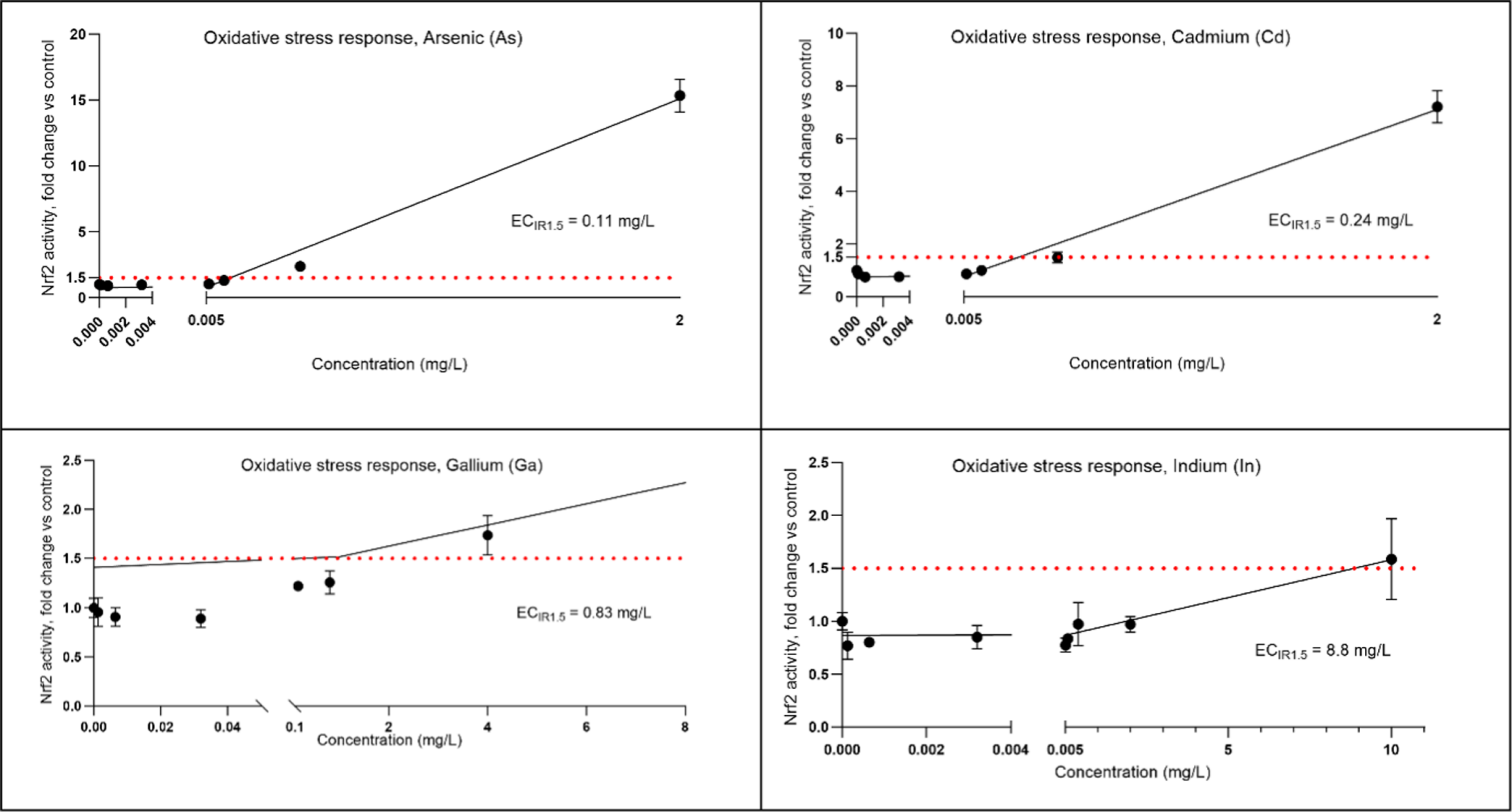
Oxidative stress response (Nrf2 activity), measured in the MCF7AREc32 cell line. Note that the scales for the different concentration–response curves differ on both the *x*- and *y*-axes, and that only elements with Nrf2 activities exceeding EC_IR1.5_ (As, Cd, Ga, In) are shown in the figure. Responses for all elements are shown in the Supporting Information, Figure S6A–B.

Four of the addressed elements (two TMCs (As and Cd) and two TCEs (Ga and In)) were found to induce oxidative stress. This effect was measured using the MCF7AREc32 cell line, and an Nrf2 activity exceeding a 1.5 induction ratio (EC_IR1.5_) was considered as indicative of the element’s potential to cause oxidative stress. Values of EC_IR1.5_ were also used to compare the toxicity between elements, as lower values, similar to lower IC values, indicate greater potency. Observing Figures 2 and S6A–B, it becomes evident that As and Cd are potent inducers of Nrf2 activity, while Pb is not at the tested concentrations. Values of EC_IR1.5_ for As and Cd were as low as 0.11 and 0.24 mg/L, respectively. Comparative EC_IR1.5_ in the literature have been challenging to locate. However, Cd and As in our study demonstrated a potency comparable to, or even higher than, the positive control substance, tert-butylhydroquinone, tBHQ (EC_IR1.5_ = 0.27 mg/L, Figure S1A), which is well-known for its pronounced propensity to trigger oxidative stress. In the Supporting Information we elaborate on the Nrf2 pathway and its role in cellular defense against oxidative stress, highlighting Nrf2 as a key regulator that mitigates oxidative damage. While our study does not reveal the specific cellular processes underlying Nrf2 activation, it is generally understood that metal-induced oxidative stress often results from the uncontrolled production of reactive oxygen species (ROS).^18,74,80−82^ The ROS are free radicals (e.g., OH°, H_2_O_2_, O_2_°^–^) that due to unpaired electrons have a high reactivity.^82^ Consequently, they play crucial roles in initiating cellular injury that can lead to e.g., adverse effects on DNA, carcinogenicity, teratogenicity, cardiovascular diseases, neurodegenerative diseases, diabetes,^83−85^ or as expressed by Ngo and Duennwald,^84^ “nearly all major human diseases”.

The TCE closest to As and Cd in terms of inducing oxidative stress, also with a distinct potency, and with an EC_IR1.5_ of 0.83 mg/L, was Ga. Activity was also observed for In, albeit at a higher EC_IR1.5_ (8.8 mg/L).

#### 3.3.1 Gallium and Indium

There are indications from previous research that both Ga and In can induce oxidative stress, in accordance with our results. Chitambar^65^ noted that exposing human lymphoma CCRF-CEM cells to gallium nitrate led to the generation of ROS, and Bériault et al.^64^ directly linked Ga to ROS production in a study involving *Pseudomonas fluorescens*. Regarding In, Lee et al.^86^ proposed oxidative stress as a possible mechanism for sperm damage in their study involving 12-week-old male Sprague–Dawley rats exposed to indium acetate. Furthermore, the generation of ROS and oxidative stress have been suggested to cause lung toxicity in human lung epithelial (A549) cells following indium oxide exposure.^87^

#### 3.3.2 Remaining TCEs (Nd, Yb, Gd, Ge, Ta, Te, Tl)

Although the majority of the investigated TCEs did not produce an oxidative stress response, as inferred from the measured Nrf2 activity in this study, there are still examples in the scientific literature suggesting otherwise. For instance, it has been proposed as an underlying mechanism for liver, brain, and spleen toxicity following oral, intraperitoneal, and abdominal administration of NdCl_3_ in mice.^88−90^ The previously mentioned Dai et al.^76^ study observed increased levels of ROS upon Yb exposure, indicating oxidative stress, and Liu et al.^91^ too found elevated levels of ROS in human hepatic cells exposed to Yb^3+^, as well as after exposure to Gd^3+^. For Ge, an increase in ROS generation, likely associated with elevated intracellular calcium levels, has previously been suggested to underlie inflammatory responses,^22^ and for Te, Roy and Hardej^62^ did not exclude the possibility that oxidative stress could underlie the observed toxicity in the astrocyte-study (see Section 3.2). The ability of Te to induce oxidative stress was also described in the previously mentioned review article by Ashraf et al.,^25^ as well as in a review article by Wei et al.^92^ The Wang et al.^75^ study, also mentioned in Section 3.2, showed indications that oxidative stress is involved in the damage to osteoblasts upon Ta exposure. But in another similar study,^93^ in which the effect of Ta nanoparticles on macrophages was investigated, the generation of ROS was found to be negligible. Tantalum nanoparticles were therefore described as both inert, nontoxic, and noninflammatory. In a study by Eskandari et al.,^94^ ROS production in isolated rat liver mitochondria was observed as a result of Tl exposure, particularly evident at the highest tested concentrations of 20–40 mg/L (0.1–0.2 mmol/L). The highest reported Tl concentration in our study was 2 mg/L, as the highest level of 10 mg/L was excluded due to cytotoxicity masking. Therefore, it cannot be ruled out that oxidative stress could have been triggered in our study if higher concentrations had been tested.

#### 3.3.3 Final Reflections

In summary, our study reveals that some TCEs, such as Ga, Nd, and Te induce similar or even stronger cytotoxic responses than As, and Cd in specific cell lines. While As and Cd also exhibited cytotoxic effects consistent with existing literature, their impact varied significantly across different cell lines. Notably, the VM7Luc4E2 cell line displayed unique sensitivity to TCEs, in contrast to its limited response to TMCs. These differences in cell line reactivity might be influenced by factors such as the differential binding of compounds to serum proteins in the culture medium, which affects the free concentration and bioavailability of elements.

When considering oxidative stress–a key process associated with cellular, organ, and systemic damage–our findings underscore the strong response of Ga. Equally important, though, is the lack of oxidative response observed for several other TCEs, despite previous evidence suggesting potential oxidative impacts. More research is essential to accurately map which TCEs induce oxidative stress and the magnitude of their effects. Additionally, future studies should focus on exploring the cellular mechanisms behind both cytotoxicity and Nrf2 activation, as these processes could not be specified in our study.

Cellular mechanisms of cytotoxicity and oxidative stress in As, Cd, and Pb are rather well-documented and can offer valuable guidance for future research aimed at identifying specific mechanisms in TCEs. The three TMCs included in this study all trigger oxidative stress primarily through the generation of ROS, although with varying mechanisms contributing to this ROS formation.^39,41,43,45,95^ For example, ROS-producing processes induced by As include superoxide production, hydroxyl radical formation, and lipid peroxidation.^39^ The ROS hydrogen peroxide is specifically formed after As exposure as a result of mitochondrial enzyme damage and subsequent impaired cellular respiration, with cellular damage as a consequence.^95^ For Cd on the other hand, EFSA^43^ highlights that oxidative stress is triggered by the depletion of cellular antioxidants in addition to ROS production, which disrupts redox balance and contributes to mitochondrial dysfunction. The disrupted redox balance in the cell can, in turn, affect transcription factors characterized by reactive cysteine molecules.^43^ Regarding Pb, the accumulation of δ-aminolevulinic acid (ALA) has been shown to trigger the formation of ROS, specifically hydroxyl radicals.^45^ Additionally, Pb inhibits several antioxidant enzymes, such as glutathione peroxidase, glutathione reductase, superoxide dismutase, and catalase, further compromising the cellular antioxidant defense system.^45,96^ Although the research on TCEs is far more limited, it has been suggested that some of these elements too can generate ROS production, as described under the elemental discussions above. Future research, however, is needed to investigate these processes in more detail.

## Supporting Information Available

Description of materials and methods for the *in vitro* based assays, responses for positive controls, concentration–response curves for cytotoxicity of all included elements, overview of responses in the oxidative stress assay for all included elements (PDF)
