# Supplementary material for: Cytotoxicity and Oxidative Stress Induced by Technology-Critical Elements versus Traditional Metal Contaminants: An In Vitro Bioassay Study
Source: Environ Sci Technol. 2025 Jan 6;59(2):1145–55. doi: 10.1021/acs.est.4c09710 (PMC11755709; doi:10.1021/acs.est.4c09710)
Supplement: Supplementary file 1 — es4c09710_si_001.pdf [file es4c09710_si_001.pdf]

Supporting Information

Cytotoxicity and Oxidative Stress Induced by Technology-Critical Elements *versus* Traditional Metal Contaminants: An *In Vitro* Bioassay Study

Anna Qvarforth[a,b*], Anna Augustsson[b], Michelle von Ehr[a], Geeta Mandava[a], Ilia Rodushkin[c,d], Emma Engström[c,d], Steffen Eisele[c], Johan Lundqvist[a]

[a]Department of Animal Biosciences, Swedish University of Agricultural Sciences, Box 7028, SE-750 07, Uppsala, Sweden
[b]Department of Biology and Environmental Science, Linnaeus University, Stuvaregatan 4, 392 31, Kalmar, Sweden
[c]ALS Laboratory Group, ALS Scandinavia AB, Aurorum 10, 977 75, Luleå, Sweden
[d]Division of Geosciences and Environmental Engineering, Luleå University of Technology, Laboratorievägen 14, 971 87, Luleå, Sweden

*Email: anna.qvarforth@lnu.se (A. Qvarforth)

Summary: 12 pages, including 6 figures.

Table of contents

1. Material and methods for the *in vitro* based methods 3

1.1. Cell culturing and maintenance 3

1.2. Sample preparation 4

1.3. Cell viability assessment 4

1.4. Nrf2 reporter gene assay 4

2. Positive controls 6

Figure S1A. Positive control (DMSO) in the cytotoxicity assays. 6

Figure S1B. Positive control (tert-butylhydroquinone, tBHQ) in the Nrf2 activity assay. 6

3. Cytotoxicity, concentration response curves 7

Figure S2. MCF7AREc32 cell line. 7

Figure S3. EcoScreen GR-KO-M1 cell line. 8

Figure S4. DR-EcoScreen cell line. 9

Figure S5. VM7Luc4E2 cell line. 10

4. Oxidative stress, overview 11

Figure S6A–B. Oxidative stress, MCF7AREc32 cell line 11

5. References 12

1. Material and methods for the *in vitro* based methods

1.1. Cell culturing and maintenance

1.1.1. Cell cultures and medium

MCF7AREc32, a stable human mammary cell line MCF7 transfected with copies of the rat GST antioxidant responsive element (ARE) linked to a luciferase gene AREc32, was kindly obtained from R. Wolf (University of Dundee, Nethergate, Scotland). The DMEM GlutaMax medium with 4.5 g/L D-glucose in which the cells were cultured, was supplemented with 10% FBS, 1% penicillin-streptomycin, and 0.8 mg/mL of Geneticin (G418) (Invivogen). The latter was not added to the experimental medium; otherwise, the two media were similar.

The **AR-EcoScreen GR-KO-M1** consists of stably transfected Chinese hamster ovary (CHO) cell line AR-EcoScreen with glucocorticoid receptor knockout mutant 1 (GR-KO M1) (JCRB, Osaka, Japan). Cell culture medium was composed of Dulbecco's Modified Eagle Medium: Nutrient Mixture F-12 (DMEM-F12 medium, Sigma-Aldrich, Irvine, United Kingdom) supplemented with 10% fetal bovine serum (FBS), 1% penicillin-streptomycin (100 units/mL penicillin, 100 µg/mL streptomycin), 1% L-glutamine, 50 µg/mL Zeocin (Invitrogen, CA, USA) and 25 µg/mL Hygromycin B Gold (InvivoGen, CA, USA). The experimental medium consisted of DMEM-F12 (Sigma-Aldrich), 10% dextran charcoal treated FBS, 1% penicillin-streptomycin and 1% L-glutamine.

The **VM7Luc4E2 cells**, from a human breast cancer cell line, were kindly received from M. Denison (University of California, CA, USA). The cell line was maintained in cell culture media containing Roswell Park Memorial Institute 1640 (RPMI 1640) with L-glutamine, 8% FBS, 0.9% penicillin-streptomycin, and the positive selector Gentamicin at a concentration of 0.55 mg/mL (Gentamicin Sulfate, Lonza). When conducting the assays, the culture medium was changed 48–72 hours before the experiment to estrogen-free medium. The experimental medium contained DMEM with 4.5 g/L glucose, 4.5% dextran-charcoal-treated FBS, 1.9% L-glutamine, 0.9% penicillin-streptomycin, and 0.38 mg/mL Gentamicin (GIBCO).

Finally, the **DR-EcoScreen cells**, from a mouse hepatoma cell line, were obtained from the Japanese Collection of Research Bioresources Cell Bank (JCRB, Osaka, Japan). The culture medium used for these cells comprised Minimum Essential Medium α (α-MEM), 5% FBS, 1% penicillin-streptomycin, and 150 µg/mL Hygromycin B Gold. The experimental medium contained no Hygromycin but was otherwise identical to the culture medium.

All supplements and mediums were obtained from Gibco, Thermo Fisher Scientific, unless stated otherwise.

1.1.2. Cell culture maintenance

The cell lines were maintained as monolayers in flasks placed in a humidified incubator at 37 ˚C and 5% CO_2. The medium was changed every second to third day. All cell lines used 0.05% Trypsin-EDTA (Gibco, Thermo Fisher Scientific) during passaging of cells. The cell lines were not used beyond passage 30.

1.2. Sample preparation

All the elements were dissolved in either 5% or 2% nitric acid, or in water, and the concentrations ranged from 0.00128 to 100 mg/L (Ga, Nd, Pb, Yb) and 0.000128 to 10 mg/L (for the remaining elements). All the dilutions were stored at -20 °C until the bioassays. All experiments included a solvent control equivalent to the solvent concentration in the samples. Vehicle solvent controls were tested in 8 replicates, while the samples were tested in four replicates. The vehicle control, 5% nitric acid, did not cause any cytotoxicity and did not exceed 1%.

1.3. Cell viability assessment

All four cell lines were tested for cell viability. Applied method was the MTS (Cell Titer 96® AQueous One Solution Cell Proliferation Assay, Promega) test for all cell lines except for the VM7Luc4E2 where instead the ATPase (CellTiter-Glo® Luminescent Cell Viability Assay, Promega) test was used. The MTS test is based on the principle that an MTS reagent containing tetrazolium is converted by viable cells into the colored product formazan. The formazan product, thus proportional to the number of viable cells, can be quantified by absorbance at 490 nm. In the ATPase assay, the viability of VM7Luc4E2 cells is quantitatively evaluated using CellTiter-Glo. This assay relies on a luminescent signal generated by CellTiter-Glo, which is directly proportional to the quantity of adenosine triphosphate (ATP) present in viable cells.

Cells were seeded in transparent 384-well plates (Costar® Corning) at specific densities: 5,200 cells/well for MCF7AREc32 and 4,000 cells/well for AR-EcoScreen GR-KO M1 and DR-EcoScreen. Following seeding, the plates were incubated for 24 hours, after which the cells were exposed to TCE/TMC samples at eight different concentrations, vehicle controls, and the positive control (10% DMSO). After an additional 24-hour incubation period, the medium was removed, and each well was treated with Earl's Balanced Salt Solution (EBSS). Subsequently, 10 µL of CellTiter 96® AQueous Reagent was added to each well, and the plates were incubated for approximately 30 minutes at 37 ˚C with 5% CO_2 before measuring absorbance. Cell viability was assessed using a Spark Multimode Microplate Reader (TECAN Spark® Multimode Microplate Reader; TECAN, Austria GmbH, SparkControl version 3.1 software).

As for the VM7Luc4E2 cells, they were seeded in white 384-well plates from Corning with a seeding density of 16,000 cells per well and incubated for 24 hours. After this initial incubation, the cells were exposed to TCEs/TMCs, vehicle controls, and the positive control (10% DMSO) for an additional 24 hours. Following the exposure period, 25 µL of CellTiter-Glo® was added to each well and allowed to incubate for approximately 15 minutes on a plate shaker to induce lysis. This was followed by a two-minute incubation without shaking. Absorbance was then measured using the Spark® Multimode Microplate Reader.

1.4. Nrf2 reporter gene assay

Nuclear factor erythroid 2-related factor 2 (Nrf2) is a crucial regulator of the cell's response to oxidative stress. As described by Escher et al.[1] and Ngo and Duennwald,[2] this regulation is carried out through the modulation of various genes responsible for antioxidants and phase II enzymes.

This regulatory process is influenced by Kelch-like ECH-associated protein (Keap1), which acts as a sort of "brake". Keap1 interacts with Nrf2 in the cell's cytosol, initiating a process called polyubiquitination, facilitated by the Cullin 3 (Cul3) E3 ubiquitin ligase. This process ultimately leads to the degradation of Nrf2, maintaining low basal levels under normal conditions. When the cell encounters oxidative stress, however, specific changes occur in Keap1, preventing it from promoting the breakdown of Nrf2. As a result, Nrf2 becomes stable, accumulates, and translocates to the nucleus. Inside the nucleus, Nrf2 interacts with so-called sMaf proteins and binds to antioxidant response elements (AREs), subsequently activating the expression of genes encoding enzymes that detoxify reactive oxygen species (ROS) and other harmful oxidants.

Similar to the cell viability assays, MCF7AREc32 cells were initially seeded in white 384-well plates (Corning) at the same concentration mentioned earlier, i.e., 5,200 cells per well. The following day, the cells were exposed to TCE/TMC samples, vehicle controls, positive controls, and 0.78 to 25 µM of tert-butylhydroquinone (tBHQ) as a reference compound for a duration of 24 hours. After exposure, the medium was removed, and the cells were lysed with 10 µL/well of Passive Lysis Buffer (PLB, Promega). The plates were then placed on a plate shaker for approximately 15–20 minutes. Once the plates were transferred to the plate reader, 10 µL/well of Luciferase reagent, LAR (Promega), was automatically injected. The results, reflecting activities for the studied endpoint, were exported to Excel files for further data processing. The *in vitro* testing was conducted using 8 non-cytotoxic concentrations in quadruplicates to ensure high precision and accurate calculation of standard deviation. Each decrease in concentration corresponded to a fivefold dilution.

2. Positive controls

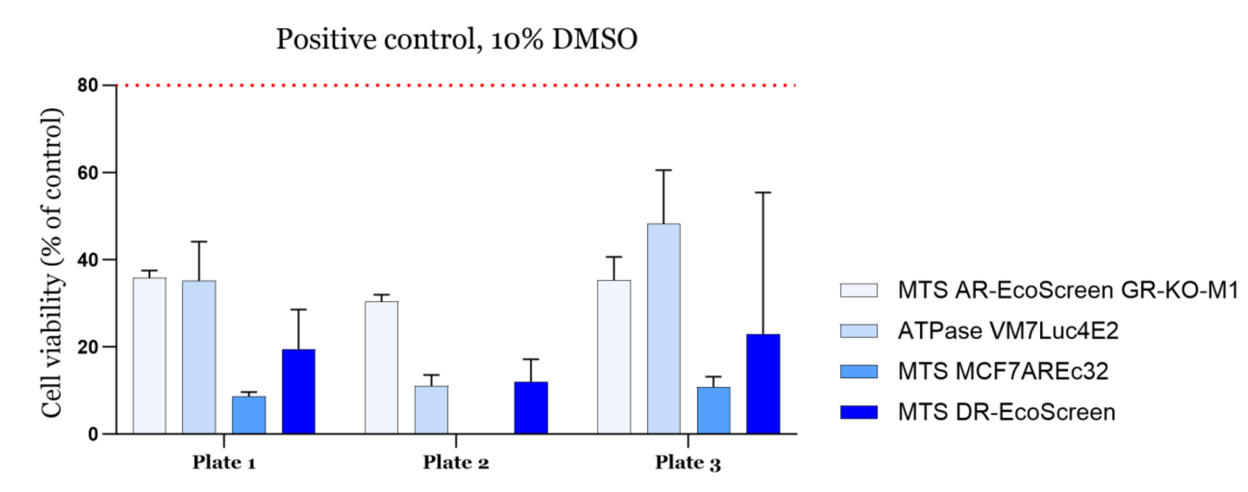

Figure S1A. *Positive control (DMSO) in the cytotoxicity assays.*
The red dotted line indicates the cut-off value for cytotoxicity, defined as a cell viability < 80% compared to the vehicle control. Data presented as mean ± standard deviation, n=4.

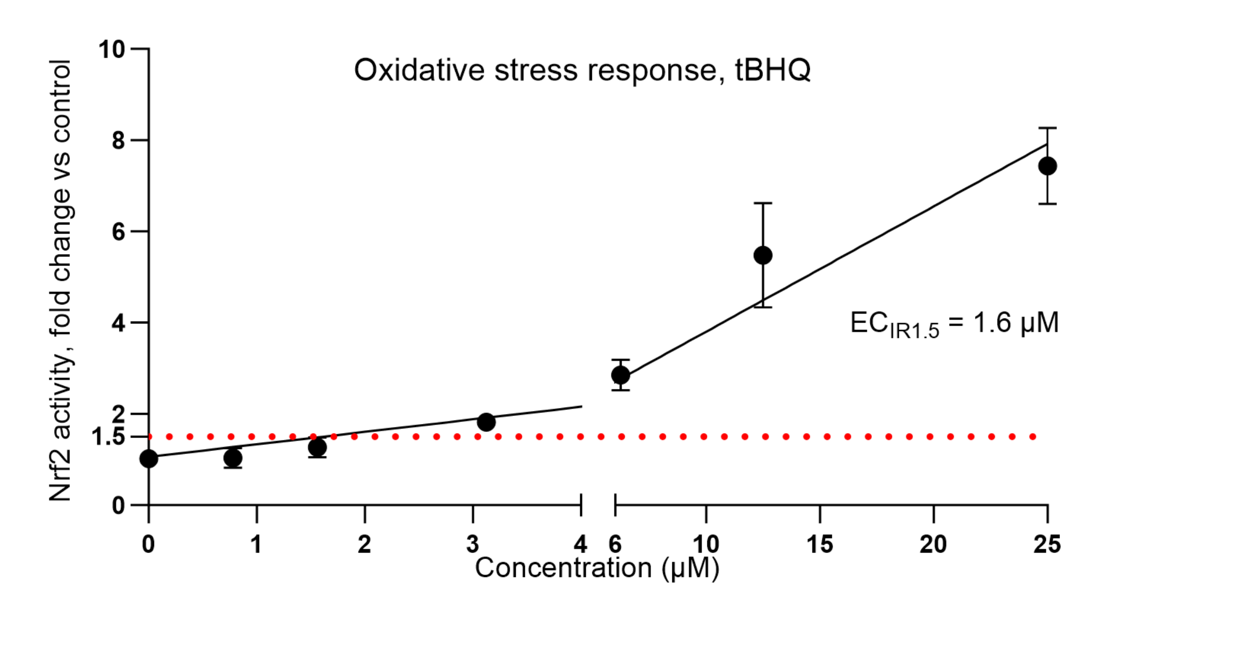

Figure S1B. *Positive control (tert-butylhydroquinone, tBHQ) in the Nrf2 activity assay.*
The red dotted line indicates the cut-off value for activity, 1.5 fold increase vs vehicle control.

3. Cytotoxicity, concentration response curves

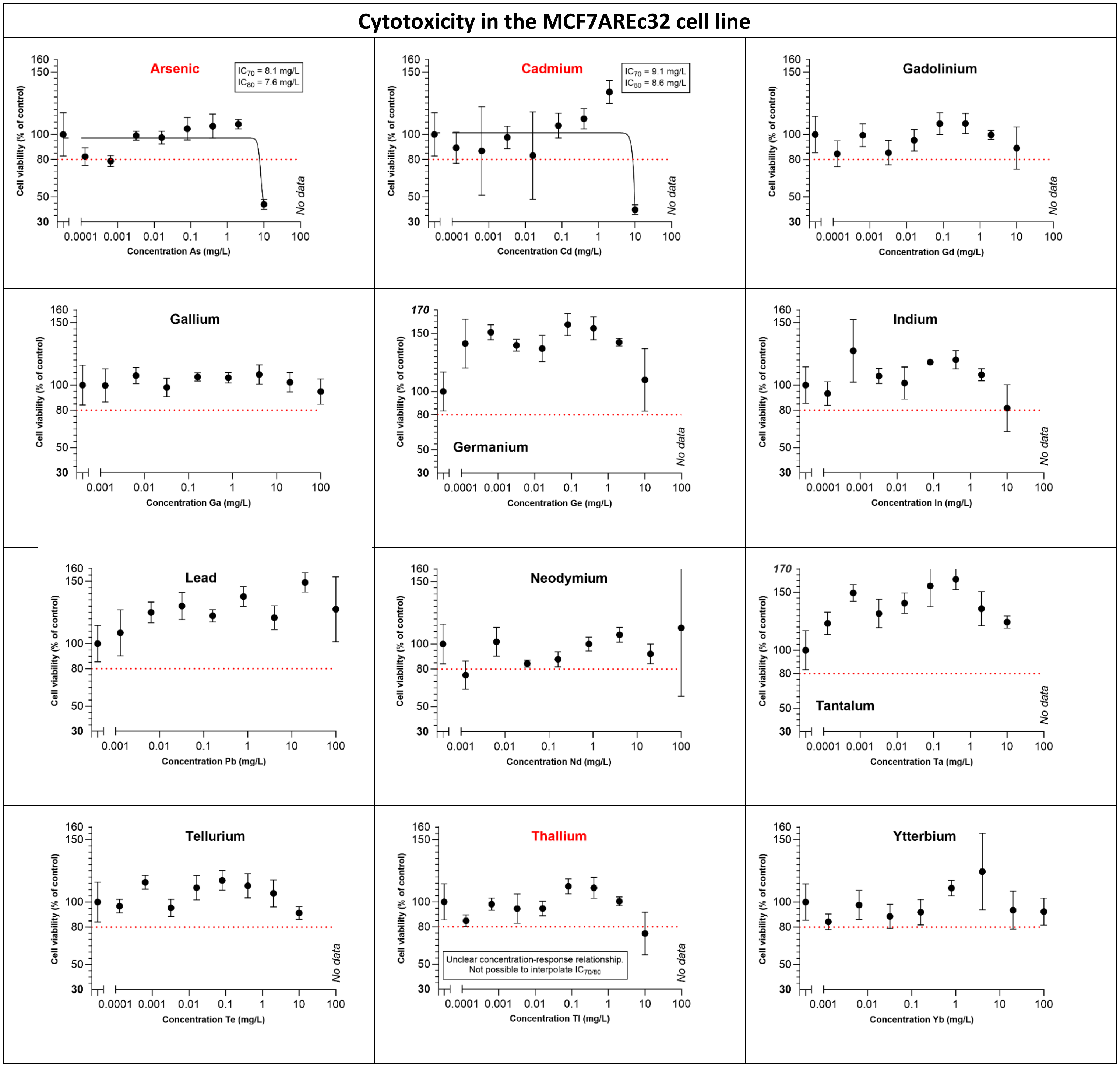

Figure S2. MCF7AREc32 cell line.
Cytotoxicity analysed with MTS test. The red dotted line indicates the cut-off value for cytotoxicity, defined as a cell viability < 80% compared to the vehicle control. For each assessed concentration, data is presented as the mean value ± standard deviation (n=4). The concentration levels for Ga, Nd, Yb, and Pb are 0.00128, 0.0064, 0.032, 0.16, 0.8, 4, 20, and 100 mg/L; and for the remaining elements 0.000128, 0.00064, 0.0032, 0.016, 0.08, 0.4, 2, and 10 mg/L. Red coloring of the elemental names points out cytotoxicity. Cytotoxicity is shown for As, Cd, and Tl. Notice that the y-axis for Ge and Ta ranges between 30 and 170%, while for the rest of the elements it ranges from 30 to 160%.

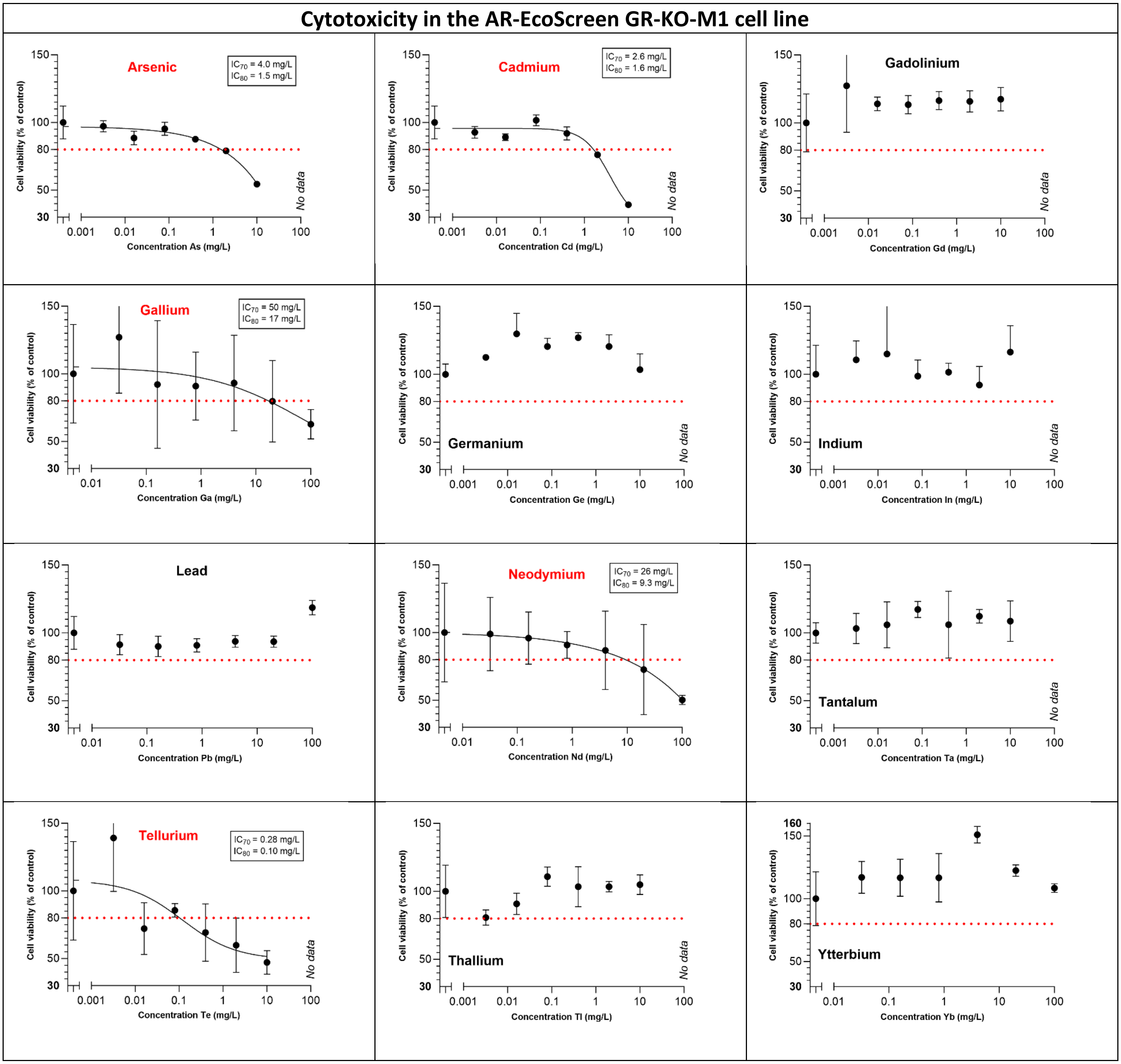

Figure S3. EcoScreen GR-KO-M1 cell line.

Cytotoxicity analysed with MTS test. The concentration levels for the elements, as well as the layout and the way cytotoxicity are defined and indicated, are consistent with **Figure S2**. Cytotoxicity is shown for As, Cd, Ga, Nd, and Te. Notice that the y-axis for Yb ranges between 30 and 160%, while for the rest of the elements it ranges from 30 to 150%.

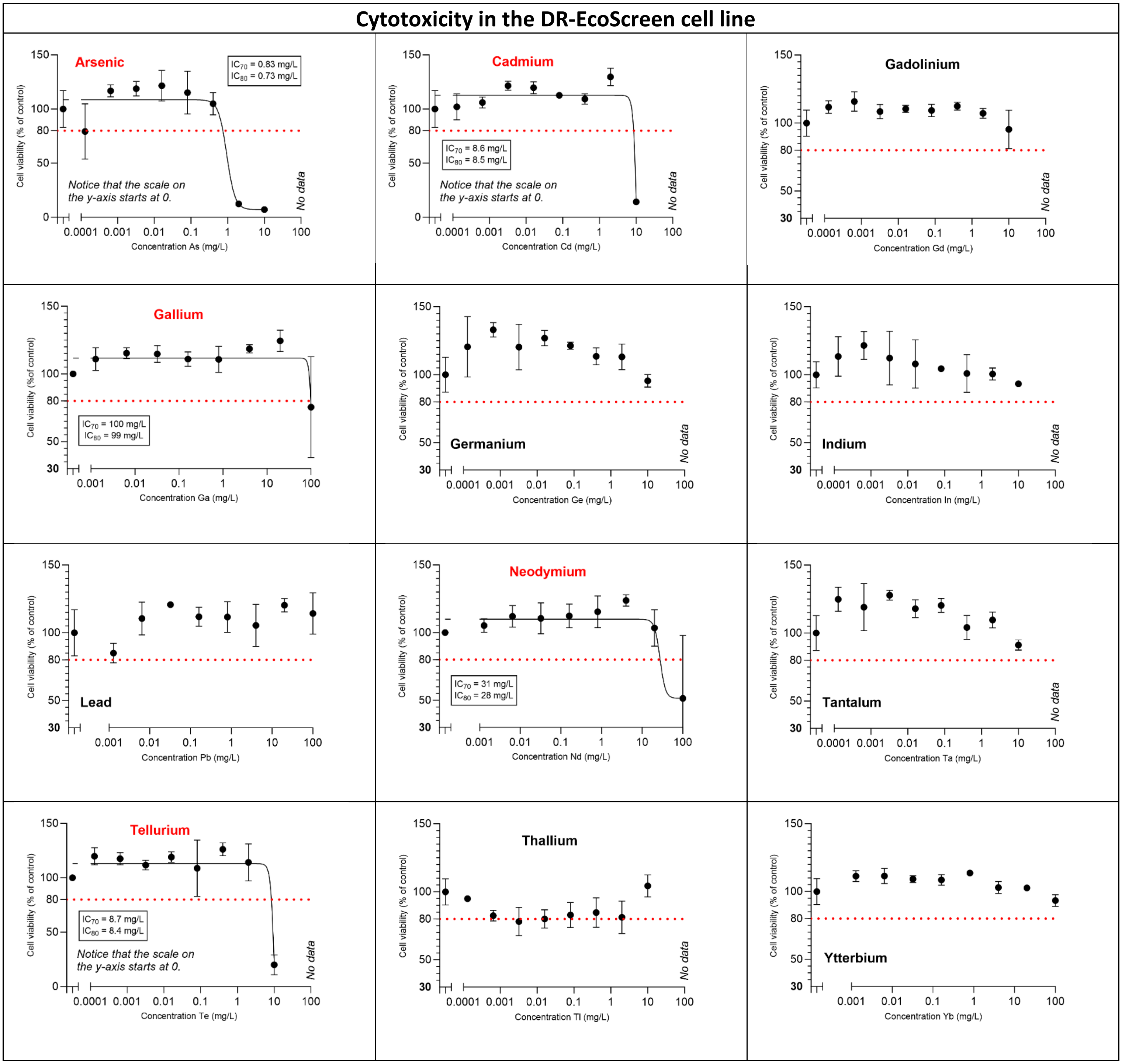

Figure S4. DR-EcoScreen cell line.

Cytotoxicity analysed with MTS test. The concentration levels for the elements, as well as the layout and the way cytotoxicity are defined and indicated, are consistent with **Figure S2**. Cytotoxicity is shown for As, Cd, Ga, Nd, and Te. Notice that the y-axis for As, Cd, and Te ranges between 0 and 150%, while for the rest of the elements it ranges from 30 to 150%.

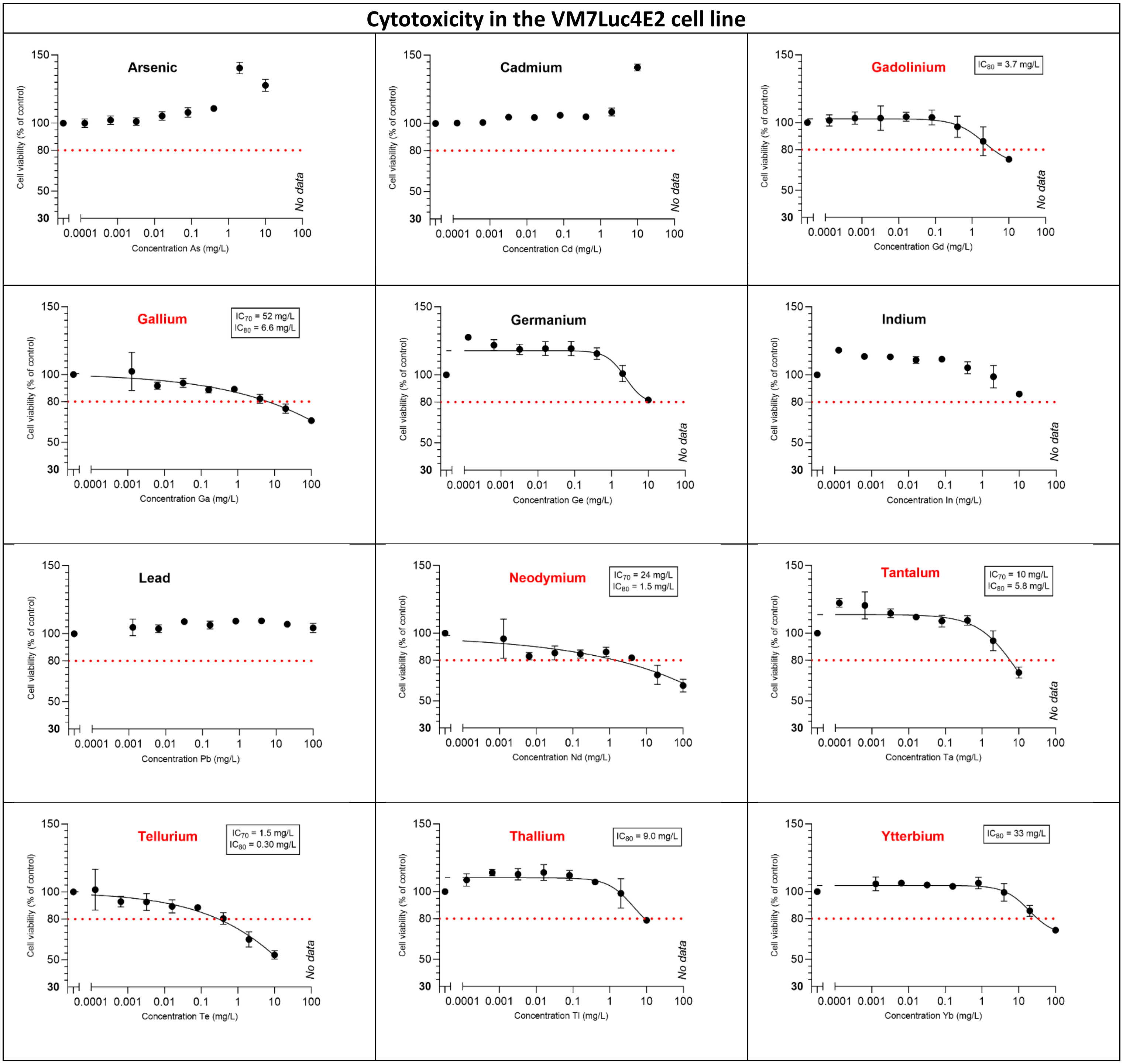

Figure S5. VM7Luc4E2 cell line.

Cytotoxicity analysed with ATPase test. The concentration levels for the elements, as well as the layout and the way cytotoxicity are defined and indicated, are consistent with **Figure S2**. Cytotoxicity is shown for Ga, Gd, Nd, Ta, Te, Tl, and Yb.

109

4. Oxidative stress, overview

Oxidative stress in the MCF7AREc32 cell line

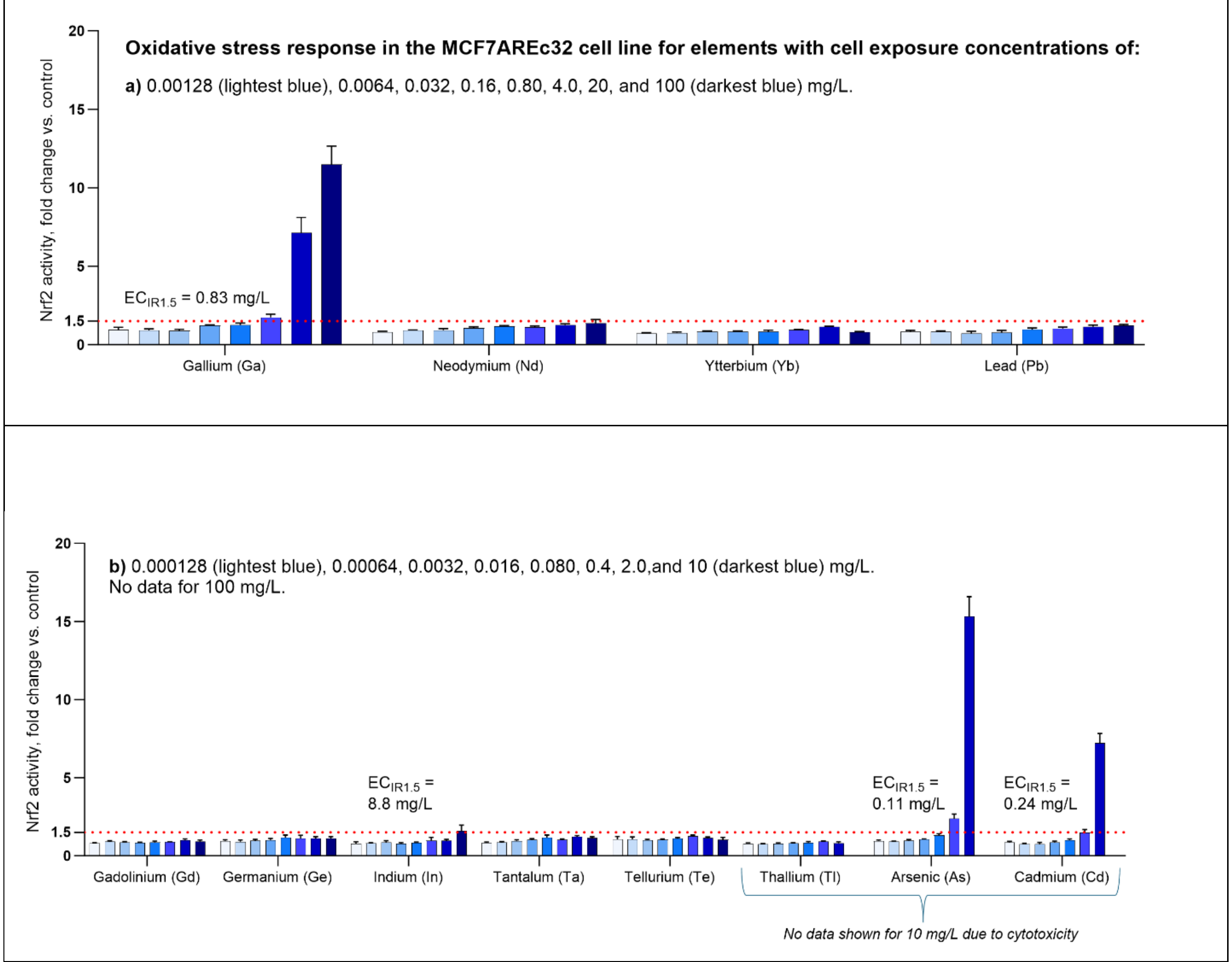

Figure S6A–B. Oxidative stress, MCF7AREc32 cell line
Oxidative stress response (Nrf2 activity), measured in the MCF7AREc32 cell line. The elements are divided in subfigures (a and b) based on the concentration in the stock solution.

5. References

(1) Escher, B.; Neale, P.; Leusch, F. *Bioanalytical Tools in Water Quality Assessment*, 2nd ed.; IWA Publishing: United Kingdom, **2021**.

(2) Ngo, V.; Duennwald, M. L. Nrf2 and Oxidative Stress: A General Overview of Mechanisms and Implications in Human Disease. *Antioxidants.* **2022**, *11*, 2345.
